# Supplementary material for: Cigarette smoke induces angiogenic activation in the cancer field through dysregulation of an endothelial microRNA
Source: Commun Biol. 2025 Mar 28;8:511. doi: 10.1038/s42003-025-07710-y (PMC11953391; doi:10.1038/s42003-025-07710-y)
Supplement: Supplementary file 1 — Supplementary Information [file 42003_2025_7710_MOESM1_ESM.pdf]

Supplemental Table S1. Characteristics of the Yale NSCLC cohort: tumor (T) samples (n=72)

**Characteristics**	**ALL-NSCLC**
Median age – yr (IQR)	69 (18)
Gender - no.(%)	
Female	42 (58.3%)
Male	30 (41.6%)
Smoking History – no.(%)	
Non-smokers	9 (12.5%)
Former smokers	50 (69.4%)
Current smokers	13 (18.1%)
Type of Procedure – no.(%)	
Surgery	40 (55.5%)
Bronchoscopy	32 (44.4%)
Time of Specimen Acquisition – no.(%)	
New diagnosis	58 (80.5%)
Established diagnosis	12 (16.6%)
Tumor recurrence	1 (1.3%)
Unable to determine	1 (1.3%)
Clinical stage – no.(%)	
Stage I	23 (31.9%)
Stage II	7 (9.7%)
Stage III	8 (11.1%)
Stage IV	26 (36.1%)
not known [A]	8 (11.1%)
Tumor Type – no.(%)	
Adenocarcinoma	39 (54.1%)
Squamous cell carcinoma	22 (30.5%)
Other [B]	11 (15.2%)
Tumor Size – no.(%) [C]	
< 3 cm	25 (34.7%)
> 3 cm	33 (45.8%)
Not available	14 (19.4%)

[A] Unable to determine the accurate clinical stage at the time of biopsy.
[B] 'Other' includes poorly differentiated carcinomas, large cell carcinoma, basaloid carcinoma, and sarcomatoid carcinoma. [C] Tumor size was determined by measuring the largest dimension in surgically resected specimens or in CT Scan images (bronchoscopic biopsy patients).

Supplemental Table S2: Characteristics of the Yale NSCLC cohort: Adjacent tissue (AT) samples (n=58)

Characteristics	ALL-NSCLC
Median age – yr (IQR)	69 (19)
Gender - no.(%)	
Female	34 (58.6%)
Male	24 (41.3%)
Smoking History - n(%)	
Non-smokers	10 (17.2%)
Former smokers	38 (52.8%)
Current smokers	10 (17.2)
Type of Procedure - n(%)	
Surgery	40 (68.9%)
Bronchoscopy	32 (55.1%)
Time of Specimen Acquisition – n(%)	
New diagnosis	49 (84.4%)
Established diagnosis	7 (12.0%)
Tumor recurrence	2 (3.4%)
Unable to determine	0 (0 %)
Clinical stage - n(%)	
Stage I	27 (46.5%)
Stage II	6 (10.3%)
Stage III	5 (8.6%)
Stage IV	12 (20.6%)
not known [A]	8 (13.7%)
Tumor Type - n(%)	
Adenocarcinoma	34 (58.6%)
Squamous cell carcinoma	8 (13.7%)
Other [B]	16 (27.5%)
Tumor Size - n(%) [C]	
< 3 cm	25 (43.1%)
> 3 cm	26 (44.8%)
Not available	7 (12.0%)

[A] Unable to determine the accurate clinical stage at the time of biopsy. [B] 'Other' includes poorly differentiated carcinomas, large cell carcinoma, basaloid carcinoma, and sarcomatoid carcinoma. [C] Tumor size was determined by measuring the largest dimension in surgically resected specimens or in CT Scan images (bronchoscopic biopsy patients).

Supplemental Table S3: Characteristics of bronchoscopic samples from smokers with NSCLC n=12

**Characteristics**	**n=12**
Median age – yr (IQR)	67 (20)
Gender - no.(%)	
Female	8 (66.7%)
Male	4 (33.3%)
Time of Specimen Acquisition –	
n(%)	9 (75%)
New diagnosis	3 (25%)
Established diagnosis	
Clinical stage - n(%)	
Stage I	1 (8.3%)
Stage II	0 (0%)
Stage III	0 (0%)
Stage IV	8 (66.7%)
not known [A]	3 (25.0%)
Tumor Type - n(%)	
Adenocarcinoma	6 (50%)
Squamous cell carcinoma	6 (50%)
Tumor Size - n(%)[B]	
< 3 cm	2 (16.7%)
> 3 cm	6 (50.0%)
Not available	4 (33.3%)

[A] Unable to determine the accurate clinical stage at the time of biopsy.
[B] Tumor size was determined by measuring the largest dimension in surgically resected specimens or in CT Scan images (bronchoscopic biopsy patients).

Supplemental Table S4: MiR-1 targets in NSCLC smokers

ARHGAP22	Rho GTPase Activating Protein 22
MOCOS	Molybdenum Cofactor Sulfurase
SH3PXD2B	SH3 And PX Domains 2B
MTL5	Testis Expressed Metallothionein Like Protein
NFRKB	Nuclear Factor Related To KappaB Binding Protein
FGD6	FYVE, RhoGEF And PH Domain Containing 6
VPS33A	VPS33A Core Subunit Of CORVET And HOPS Complexes
PUS1	Pseudouridine Synthase 1
GOLGA3	Golgin A3
SEMA4B*	Semaphorin 4B
TRAP1*	TNF Receptor Associated Protein 1
TFAP4*	Transcription Factor AP-4
XYLT2	Xylosyltransferase 2
NOTCH3*	Notch Receptor 3
NPAS2	Neuronal PAS Domain Protein 2
COL5A2*	Collagen Type V Alpha 2 Chain
CEP250*	Centrosomal Protein 250
BCAS4	Breast Carcinoma Amplified Sequence 4
CPT1B*	Carnitine Palmitoyltransferase 1B
SEC61A1*	SEC61 Translocon Subunit Alpha 1
WHSC1*	Nuclear Receptor Binding SET Domain Protein 2
HS3ST1*	Heparan Sulfate-Glucosamine 3-Sulfotransferase 1
ITGA2*	Integrin Subunit Alpha 2
SDCCAG3	Endosome Associated Trafficking Regulator 1

* Genes previously shown to be related to tumor progression in NSCLC, (1-12).

Supplemental Figure S1

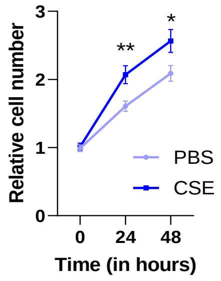

HUVECs were exposed to 10% CSE (or PBS, control) at the indicated times (in minutes) and cell numbers determined by counting using a hemocytometer. Relative cell numbers were presented after normalizing to values at time ‘0’(n>12 from 3 experiments, *p= 0.021, **p=0.0065)

Supplemental Figure S2:

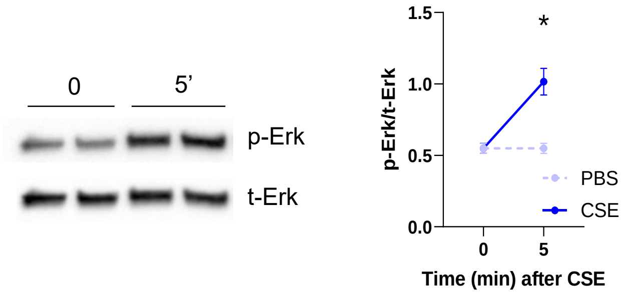

HUVECs were starved and treated with CSE for indicated time and cells were harvested for Western analysis. Graphs represent ratio of band intensities (phospho to total ERK) after normalizing with time 0 value (n=3, *p= 0.0257).

Supplemental Figure S3:

3'UTR	NOTCH3 3'UTR
Seed position	7701-7077
Type	7-mer-A1

```
5'... u c c c u c a c u u c a c u g c a u u c c a ...3'
            |     | | | | |         | | | | | |
   3' u a u G u a u g a a g a a a u g u a a g g u 5'    miR-1
```

3'UTR	HS3ST1
Seed position	1793-9
Type	7-mer-m8

```
5'... g g u C a c a g u u u u a c u c a u u c c c ...3'
            | |   |   | |   |       | | | | | |
   3' u a u g u a u g a a g a a a u g u a a g g u 5'    miR-1
```

Coding region	SEMA4B
Seed position	1017-1025
Type	8-mer

```
5'... u g u g g c c u c a g c c u a c a u u c c u ...3'
                                | | | | | | | |
   3' u a u g u a u g a a g a a a u g u a a g g u 5'    miR-1
```

Coding region	TFAP4
Seed position	375-381
Type	8-mer

```
5'... c u g u a g c c u u g c c a a c a u u c c a ...3'
        |     |     | | |         | | | | | | |
   3' u a u g u a u g a a g a a a u g u a a g g u 5'    miR-1
```

MiR-1 targets (A) NOTCH3 (B) HS3ST1 (C) SEMA4B (D) TFAP4 in this study. Predicted miR-1 binding sites are depicted in green in target sequence and corresponding nucleotides in miR-1 mature sequence are in red. Vertical lines shows complementary bases.

Supplementary Figure S4: Uncropped Gel #2 for Figure 6B (used in the main figure and analysis)

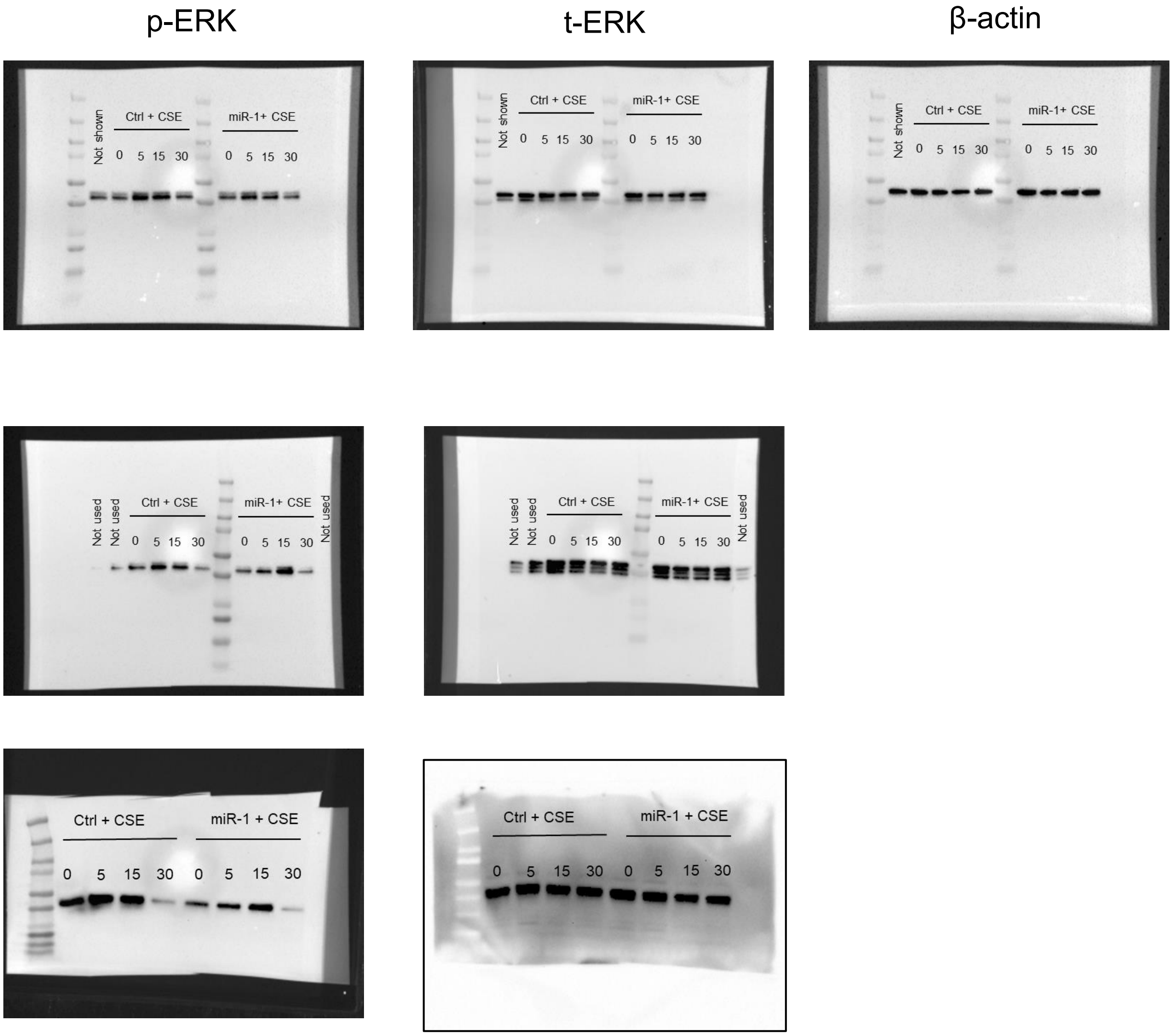

Supplemental Figure S5: **Uncropped western blots for Figure S2 (used in the supplemental figure and analysis)**

p-ERK

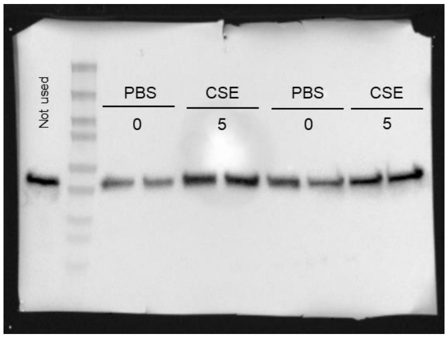

t-ERK

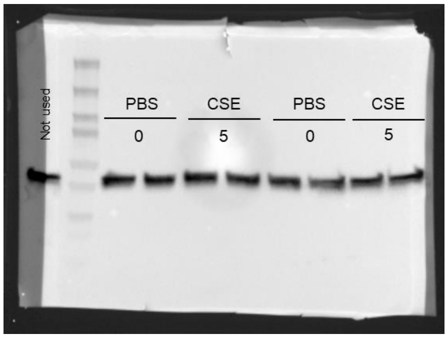

References

1. Jiang J, Lu Y, Zhang F, Pan T, Zhang Z, Wan Y, Ren X, and Zhang R. Semaphorin 4B promotes tumor progression and associates with immune infiltrates in lung adenocarcinoma. *BMC cancer.* 2022;22(1):632.
2. Li X, Li X, Chen S, Wu Y, Liu Y, Hu T, Huang J, Yu J, Pei Z, and Zeng T. TRAP1 shows clinical significance in the early diagnosis of small cell lung cancer. *Journal of Inflammation Research.* 2021:2507-14.
3. Shen Q, Xu Z, Sun G, Wang H, and Zhang L. TFAP4 activates IGF2BP1 and promotes progression of non–small cell lung cancer by stabilizing TK1 expression through m6A modification. *Molecular Cancer Research.* 2022;20(12):1763-75.
4. Xiu M, Wang Y, Li B, Wang X, Xiao F, Chen S, Zhang L, Zhou B, and Hua F. The role of Notch3 signaling in cancer stemness and chemoresistance: molecular mechanisms and targeting strategies. *Frontiers in Molecular Biosciences.* 2021;8(694141.
5. Zhang H, Liu R, Zhang B, Huo H, and Song Z. Advances in the study of circadian genes in non-small cell lung cancer. *Integrative Cancer Therapies.* 2022;21(15347354221096080.
6. Tammela T, Sanchez-Rivera FJ, Cetinbas NM, Wu K, Joshi NS, Helenius K, Park Y, Azimi R, Kerper NR, and Wesselhoeft RA. A Wnt-producing niche drives proliferative potential and progression in lung adenocarcinoma. *Nature.* 2017;545(7654):355-9.
7. Guijo M, Ceballos-Chávez M, Gómez-Marín E, Basurto-Cayuela L, and Reyes JC. Expression of TDRD9 in a subset of lung carcinomas by CpG island hypomethylation protects from DNA damage. *Oncotarget.* 2018;9(11):9618.
8. Ye Z, Zhang H, Kong F, Lan J, Yi S, Jia W, Zheng S, Guo Y, and Zhan X. Comprehensive analysis of alteration landscape and its clinical significance of mitochondrial energy metabolism pathway-related genes in lung cancers. *Oxidative medicine and cellular longevity.* 2021;2021(
9. Guan Q, Zhao P, Tian Y, Yang L, Zhang Z, and Li J. Identification of cancer risk assessment signature in patients with chronic obstructive pulmonary disease and exploration of the potential key genes. *Annals of Medicine.* 2022;54(1):2308-19.
10. Denys A, and Allain F. The emerging roles of heparan sulfate 3-O-sulfotransferases in cancer. *Frontiers in oncology.* 2019;9(507.
11. Chen J, Gao C, and Zhu W. Long non-coding RNA SLC25A25-AS1 exhibits oncogenic roles in non-small cell lung cancer by regulating the microRNA-195-5p/ITGA2 axis. *Oncology letters.* 2021;22(1):1-13.
12. García-Carpizo V, Sarmentero J, Han B, Graña O, Ruiz-Llorente S, Pisano DG, Serrano M, Brooks HB, Campbell RM, and Barrero MJ. NSD2 contributes to oncogenic RAS-driven transcription in lung cancer cells through long-range epigenetic activation. *Scientific reports.* 2016;6(1):32952.
