# Supplementary material for: Cigarette smoke induces angiogenic activation in the cancer field through dysregulation of an endothelial microRNA
Source: Commun Biol. 2025 Mar 28;8:511. doi: 10.1038/s42003-025-07710-y (PMC11953391; doi:10.1038/s42003-025-07710-y)
Supplement: Supplementary file 2 — Description of Additional Supplementary Materials [file 42003_2025_7710_MOESM2_ESM.pdf]

Description of Additional Supplementary Files

File name: Supplementary Data 1

Description: Values for all data points found in graphs
