# Supplementary material for: Cigarette smoke induces angiogenic activation in the cancer field through dysregulation of an endothelial microRNA
Source: Commun Biol. 2025 Mar 28;8:511. doi: 10.1038/s42003-025-07710-y (PMC11953391; doi:10.1038/s42003-025-07710-y)
Supplement: Supplementary file 4 — Reporting Summary [file 42003_2025_7710_MOESM4_ESM.pdf]

nature portfolio

Corresponding author(s): Shervin Seyedtaghi Takyar

Last updated by author(s): 2-6-25

Reporting Summary

Nature Portfolio wishes to improve the reproducibility of the work that we publish. This form provides structure for consistency and transparency in reporting. For further information on Nature Portfolio policies, see our Editorial Policies and the Editorial Policy Checklist.

Statistics

For all statistical analyses, confirm that the following items are present in the figure legend, table legend, main text, or Methods section.

n/a	Confirmed	
☐	☒	The exact sample size (*n*) for each experimental group/condition, given as a discrete number and unit of measurement
☐	☒	A statement on whether measurements were taken from distinct samples or whether the same sample was measured repeatedly
☐	☒	The statistical test(s) used AND whether they are one- or two-sided<br>*Only common tests should be described solely by name; describe more complex techniques in the Methods section.*
☐	☒	A description of all covariates tested
☐	☒	A description of any assumptions or corrections, such as tests of normality and adjustment for multiple comparisons
☐	☒	A full description of the statistical parameters including central tendency (e.g. means) or other basic estimates (e.g. regression coefficient) AND variation (e.g. standard deviation) or associated estimates of uncertainty (e.g. confidence intervals)
☐	☒	For null hypothesis testing, the test statistic (e.g. *F*, *t*, *r*) with confidence intervals, effect sizes, degrees of freedom and *P* value noted<br>*Give P values as exact values whenever suitable.*
☒	☐	For Bayesian analysis, information on the choice of priors and Markov chain Monte Carlo settings
☒	☐	For hierarchical and complex designs, identification of the appropriate level for tests and full reporting of outcomes
☒	☐	Estimates of effect sizes (e.g. Cohen's *d*, Pearson's *r*), indicating how they were calculated

Our web collection on statistics for biologists contains articles on many of the points above.

Software and code

Policy information about availability of computer code

Data collection	Data collection for the clinical cohort was performed by manual chart review and was deposited in an Excel file.
Data analysis	Statistical analysis was performed using R statistical package in the R statistical language and environment (R-project.org; version 3.5.1) and GraphPad Prism software version 10.1.2. No custom software was used in this manuscript.

For manuscripts utilizing custom algorithms or software that are central to the research but not yet described in published literature, software must be made available to editors and reviewers. We strongly encourage code deposition in a community repository (e.g. GitHub). See the Nature Portfolio guidelines for submitting code & software for further information.

Data

Policy information about availability of data

All manuscripts must include a data availability statement. This statement should provide the following information, where applicable:

- Accession codes, unique identifiers, or web links for publicly available datasets
- A description of any restrictions on data availability
- For clinical datasets or third party data, please ensure that the statement adheres to our policy

RNA-sequencing data were deposited in the NCBI GEO database under accession number GSE239928. Values for all data points found in graphs can be found in the 'supporting data values' file and will be available from the corresponding author upon request.

Research involving human participants, their data, or biological material

Policy information about studies with human participants or human data. See also policy information about sex, gender (identity/presentation), and sexual orientation and race, ethnicity and racism.

Reporting on sex and gender	Sex has been reported and analyzed as a covariate. Data on gender has not been available in the dataset and not reported.
Reporting on race, ethnicity, or other socially relevant groupings	Data on race and ethnicity were not available in the dataset and not reported.
Population characteristics	Population characteristics and exclusion/criteria for the Yale NSCLC cohort have been described in Methods under the title: "Patient characteristics, inclusion criteria, and clinical data collection". Population characteristics for the PROPECT cohort has ben published previously and the relevant reference has been mentioned in the Methods section under the title "MiR-1 measurement in PROSPECT cohort".
Recruitment	The recruitment exclusion criteria are mentioned in Methods section under "Patient characteristics, inclusion criteria, and clinical data collection" heading.
Ethics oversight	The protocols were approved by the Yale University Institutional Review Board and HIC numbers are included in the Methods section.

Note that full information on the approval of the study protocol must also be provided in the manuscript.

Field-specific reporting

Please select the one below that is the best fit for your research. If you are not sure, read the appropriate sections before making your selection.

☒ Life sciences ☐ Behavioural & social sciences ☐ Ecological, evolutionary & environmental sciences

For a reference copy of the document with all sections, see nature.com/documents/nr-reporting-summary-flat.pdf

Life sciences study design

All studies must disclose on these points even when the disclosure is negative.

Sample size	Sample sizes were chosen based on previous data and pilot experiments.
Data exclusions	No data was excluded from analysis
Replication	A minimum of three replicates were used for each experiments.
Randomization	Randomization was not relevant for our studies.
Blinding	Blinding was not relevant for correlation studies. Investigators were not blinded to the group allocations in the biochemistry studies in the lab.

Reporting for specific materials, systems and methods

We require information from authors about some types of materials, experimental systems and methods used in many studies. Here, indicate whether each material, system or method listed is relevant to your study. If you are not sure if a list item applies to your research, read the appropriate section before selecting a response.

Materials & experimental systems

n/a	Involved in the study	
☐	☒	Antibodies
☐	☒	Eukaryotic cell lines
☒	☐	Palaeontology and archaeology
☐	☒	Animals and other organisms
☐	☒	Clinical data
☒	☐	Dual use research of concern
☒	☐	Plants

Methods

n/a	Involved in the study	
☒	☐	ChIP-seq
☒	☐	Flow cytometry
☒	☐	MRI-based neuroimaging

Antibodies

Antibodies used	Total Erk: #9102, P-Erk1/2 (Thr202/Tyr204): #9101 from Cell Signaling, Ago-2 clone 11A9 #SAB4200085 Sigma-Aldrich
Validation	Signaling antibodies were validated based on the size of the bands on the Western blot and the changes in response to VEGF. Antibodies were used as per the manufacturer's protocol

Eukaryotic cell lines

Policy information about cell lines and Sex and Gender in Research

Cell line source(s)	Cell lines were purchased from ATCC.
Authentication	EAhy926 cells were purchased from ATCC and not authenticated while performing the experiments
Mycoplasma contamination	Cell lines , Human pulmonary microvascular cells, and HUVEC were purchased from ATCC, Lonza or isolated in lab and Vascular Biology Therapeutics (VBT) at Yale, respectively. All cells were tested visually for any bacterial contamination but none of the cells are tested specifically for mycoplasma contamination.
Commonly misidentified lines (See ICLAC register)	HPMEC= Human Pulmonary Microvascular endothelial cells

Animals and other research organisms

Policy information about studies involving animals; ARRIVE guidelines recommended for reporting animal research, and Sex and Gender in Research

Laboratory animals	6-8 week old C57Bl/6 mice
Wild animals	mice were purchased from commercial vendors. such Jackson lab.
Reporting on sex	In the murine studies, sex was not considered as a biological variable and the studies involved only female mice because of the ease of housing them together.
Field-collected samples	N/A
Ethics oversight	Institutional Animal Care & Use Committee (IACUC) at Yale University

Note that full information on the approval of the study protocol must also be provided in the manuscript.

Clinical data

Policy information about clinical studies

All manuscripts should comply with the ICMJE guidelines for publication of clinical research and a completed CONSORT checklist must be included with all submissions.

Clinical trial registration	The data presented are not from a clinical trial.
Study protocol	N/A
Data collection	N/A
Outcomes	N/A

Plants

Seed stocks	N/A
Novel plant genotypes	N/A
Authentication	N/A
